# Association between resting-state EEG oscillation and psychometric properties in perimenopausal women

**DOI:** 10.1186/s12905-022-01729-7

**Published:** 2022-05-10

**Authors:** Ren-Jen Hwang, Hsiu-Chin Hsu, Lee-Fen Ni, Hsin-Ju Chen, Yu-Sheun Lee, Yueh-O. Chuang

**Affiliations:** 1 Department of Nursing, Chang Gung University of Science and Technology, Taoyuan City, 33303 Taiwan, ROC; 2 Department of Nursing, Chang Gung Memorial Hospital, Linkou, Taoyuan City, 33303 Taiwan, ROC; 3 Graduate Institute of Gerontology and Health Care Management, Chang Gung University of Science and Technology, Taoyuan City, 33303 Taiwan, ROC; 4 Department of Internal Medicine, Chang Gung Memorial Hospital, Taoyuan City, 33303 Taiwan, ROC

**Keywords:** Electroencephalography, Premenopausal women, Psychometric properties

## Abstract

**Background:**

The perimenopausal period is associated with a higher risk of various mood disorders. Similarly, although resting-state electroencephalogram (rsEEG) brain oscillatory activity has been associated with various neuropsychological disorders and behaviours, these issues have not been assessed in perimenopausal women. This study aimed to evaluate quantitative relationships between psychometric properties and rsEEG rhythms (delta, theta, alpha, beta and gamma powers) in perimenopausal women.

**Methods:**

A cross-sectional correlational descriptive study was conducted to quantitatively analyze the correlations between rsEEG low-to-high band activities (delta, theta, alpha, beta, and gamma powers) and psychometric properties in 14 perimenopausal women. Participants completed a psychological inventory comprising the State Anxiety Inventory (SAI), Depression Inventory (DI), Behavioural Inhibition Scale (BIS) and short-form UPPS Impulsive Behaviour Scale (IS) before EEG recording.

**Results:**

Results showed that impulsivity was positively related to the beta power, symmetrical at most channels (frontal, temporal, central, parietal and occipital regions; *p* < .05); but did not related to the delta, theta, alpha and gamma powers. The brainwave low-to-high bands, delta, theta, alpha, beta and gamma power were not associated with DI, SAI or BIS scores.

**Conclusions:**

This study’s findings propose that significantly enhanced resting-state beta activity is a trait of impulsivity in perimenopausal women. Therefore, results have potential implications for the preclinical or clinical evaluation of these issues in perimenopausal women.

## Background

Perimenopause is a part of the processes of a woman’s transition into menopause that encompasses the final years of her reproductive life [1]. Stages of Reproductive Ageing Workshop (STRAW) [2, 3] divided the adult female life into three broad phases: the reproductive, menopausal transition and postmenopause, with each of these phases presenting their issues. In particular, reproductive hormone fluctuations characterise perimenopause, leading to mood disorders and other psychiatric problems [4]. Therefore, research has suggested that the stages of hormonal fluctuations during a woman’s lifespan run parallel with an increased risk of mood disturbances [5, 6]. Women between 45 and 55 years of age experience a phase of life known as “the window of vulnerability,” during which mental health problems that occur, such as anxiety and depressive symptoms, have been extensively explored [5]. Furthermore, women in the perimenopause stage frequently experience emotional changes, such as irritability, sadness, lack of motivation, fatigue and mood swings [7, 8], and mild cognitive decline associated with perimenopause has also been reported [9]. These emotions significantly influence social cognitive processes, which in turn, influence life outcomes [10, 11]. Thus, a better understanding of this life period, which every woman passes through, can be gained through the efforts of neuroscientists, including those in the biological and psychological sciences, to detect early-stage biomarkers of cognitive decline and work on the implications of brain oscillations. However, limited evidence on such oscillations has been gathered from preclinical, perimenopausal women.

Apart from the anxiety and depression experienced during perimenopause [12], negative affective behaviours, such as *impulsive behaviour strength (IS)* and *behavioural inhibition sensitivity (BIS)*, have also been reported in relation to hormonal imbalance [13–16]. Studies have shown that increasing oestrogen levels lead to less impulsive decision making [12, 15–17]. Moreover, the hormonal imbalance influences impulsivity strength, relating to aggressive behaviour, which is relevant to the serotonergic modulation in neural circuits [16–18]. Similarly, diminished brain serotonin synthesis is further linked with lack of impulse control and various psychiatric disorders [18]. Besides, ovarian hormones mediate the behavioural inhibition hypothesis that potential mechanisms occur through the dopamine pathway across the cortical limbic network [19]. The behavioural inhibition system is activated through aversive stimulation, responding to conditioned stimuli of punishment, novel stimuli and innate fear stimuli. It also elicits the affective state of anxiety and leads to behavioural inhibition [20, 21]. Therefore, the reproductive hormone is correlated with psychological alterations and symptoms that can negatively impact emotion and mood. However, to the best of our knowledge, no study has yet surveyed the resting EEG in perimenopause women. Furthermore, although resting EEG signals have been widely used in classifying different emotional states [22–24], no such signals exist for this middle age group of perimenopause women. The present study findings underline the importance of the selected psychometric properties of this study.

The characterisation of rsEEG brain oscillatory activity is a practical methodology used to study human behaviour. Clinically, EEG signals have been used as valuable indicators of psychological characteristics. rsEEG signals are composed of multiple waves cycling at different frequencies: delta (δ: 0.5–4 Hz), theta (θ: 4–8 Hz), alpha (α: 8–13 Hz), beta (β: 13–30 Hz) and gamma (γ: 31–40 Hz) [25]. Essentially, human behaviour begins in the brain. Therefore, quantitative EEG (qEEG) signals can be interpreted and clinically applied to evaluate brain function. Furthermore, a network of brain regions known as the ‘default mode network’ shows increased brain activity even in the resting state, reflecting spontaneous cognitive processes [26]. Hence, spontaneous activities and their various rhythms correlate with psychometric properties relevant to psychological factors in physical health and illness that are known to influence neuropsychiatric diseases [27, 28]. The quantitative spectral analysis of rsEEG provides an efficient, convenient and relatively inexpensive way to study the relationships between brain activity and behaviour [29]. Through observations of brain rhythms and considering their tagged functional roles, an attempt was made to reveal how activity across rsEEG frequency bands is associated with the psychological characteristics of perimenopausal women. Such research is necessary, as this potential neural entrainment is not completely covered in previous literature. Thus, examining resting-state EEG data is proposed to enhance our understanding of basic brain functions.

Self-report inventories have become the most prevalent method used to assess psychological characteristics. The psychological inventories used in this study were chosen according to common psychological factors experienced during perimenopause and the early stages of ageing [30–33]. These scales have been widely used in psychological research or clinical practise, and their validity and reliability have been well-established. These following scales are included: (1) the Center for Epidemiologic Studies-Depression scale (CES-D; Depression Inventory [DI]) was developed by the National Institute of Mental Health, measuring depressive symptoms in the general population, (2) the State-Trait Anxiety Inventory (SAI), a reflective psychological self-report inventory relating to anxiety, which was assessed across two dimensions (state and trait) [34]. State anxiety denotes negative mood, reflecting a ‘transitory emotional state or condition of the human organism’. (3) the BIS assessment attributes sensitivity to signals of punishments [35]. Heightened BIS sensitivity is proposed to increase one’s risk of developing anxiety or depressive disorders [36]. Finally, (4) the modified short version of the UPPS IS assesses impulsive behaviour, commonly related to a sense of urgency, sensation seeking and lack of premeditation and perseverance [32, 37]. Thus, four inventories—the DI, SAI, BIS and IS—were included in our study.

Taken together, this study investigated the relationship between neuropsychological assessments and quantitative rsEEG band power in perimenopause women. Subsequently, the cross-correlation between EEG frequency bands and psychometric properties examined whether potential neurophysiological trait markers existed for specific psychological characteristics in non-clinical perimenopausal women.

## Methods

### Participants and ethical considerations

The present research was a cross-sectional correlational study, and all procedures were conducted following the Institutional Review Board of CGMH, where 14 right-handed individuals aged 46 and 54 years (*M*_age_ = 51 years) participated. We recruited participants through advertisements among the community of Tao Yuan, Taiwan. All subjects were within the menopausal transition phase, characterised using the Stages of Reproductive Ageing Workshop (STRAW) [3, 14, 17]. Participants were screened via standardised interviews. Participants reported irregular menstrual cycles but were not menopausal and reported as not experiencing menstruation or blood discharge in the past year. Subsequently, participants were excluded if they (1) had used oral contraceptive pills or hormone therapy in the past year (2) had a history of neurological, psychiatric, or personality disorders, including premenstrual dysphoric disorder, as evaluated using the *Diagnostic and Statistical Manual of Mental Disorders*, Fourth Edition or (3) had auditory-visual impairment. Before the experiments, all participants were asked to refrain from consuming alcohol for at least 48 h, caffeine and tobacco for 12 h, and engaging in physical activity for 6 h. Furthermore, written informed consent was obtained from each participant before the experiment.

### EEG recording and preprocessing

**Fig. 1:**
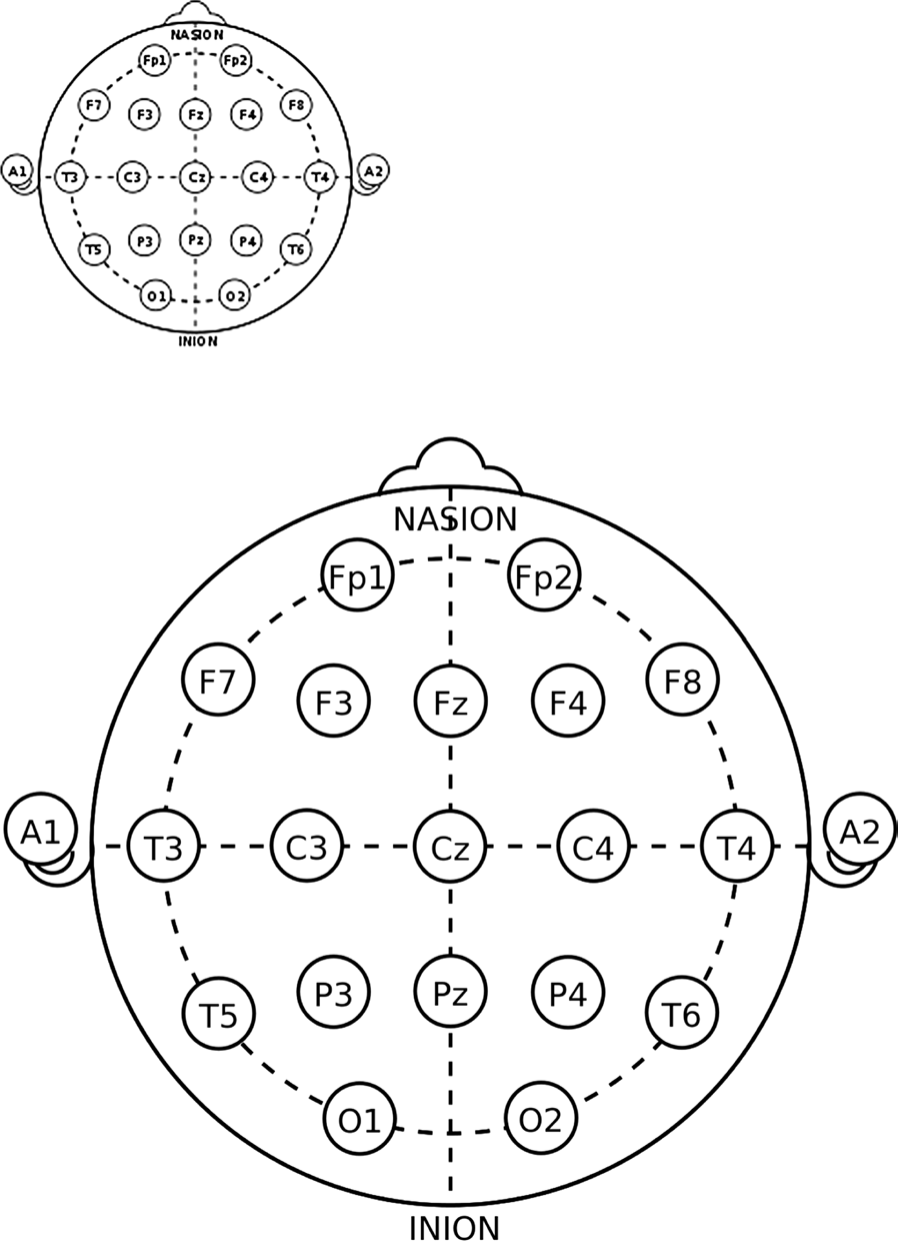
Electrode placement based on the International 10–20 System. The 19 sites: Fp1, Fp2, F3, F4, F7, F8, Fz, C3, C4, Cz, T3, T4, T5, T6, P3, P4, Pz, O1, and O2 within the standard 10–20 system, covering symmetrical brain areas; the anterofrontal, frontal, anterotemporal, temporal, posterior temporal, central, parietal, and occipital regions, are shown

Participants were tested in a light-controlled and sound-attenuated room. First, spontaneous brain activity was recorded continuously for three minutes while participants relaxed and stayed awake with their eyes closed. The study was conducted using an Encerphalan-EEGR-19/26 computerised EEG (Version 5.4–16–2.0, Medicom MTD). Then, EEG activity was recorded from 19 sites—Fp1, Fp2, F3, F4, F7, F8, Fz, C3, C4, Cz, T3, T4, T5, T6, P3, P4, Pz, O1 and O2, within the standard 10–20 system, covering symmetrical brain areas: the anterofrontal, frontal, anterotemporal, temporal, posterior temporal, central, parietal and occipital regions (Fig. 1). EEG and electrooculography were recorded at a sampling rate of 256 Hz/s, and the high-and low-frequency filters were set at 70 Hz and 0.5 Hz, respectively.

Each recording was visually screened to remove epochs with head movements and eye blinks, after which a computer-based rejection algorithm discarded any epochs with an activity greater than ± 75 μV in amplitude. Subsequently, the absolute power value (APV, μV^2^) and relative power value (RPV, %) were recorded using power spectrum analysis from non-artefact four-second signals of approximately 20–45 epochs. Then, the baseline-correction was applied to all accepted epochs, and the fast Fourier transforms were averaged in five selected frequency bands: delta (δ; 0.5–4 Hz), theta (θ; 4–8 Hz), alpha (α; 8–13 Hz), beta (β; 13–30 Hz) and gamma (γ; 31–40 Hz). Next, to minimise the effects of inter-individual variability in power, relative power (%) was obtained by computing the fraction of power in each frequency band, divided by the sum of power measurements across 0.5–40 Hz for each frequency band in each of the 19 channels. Finally, relative power was calculated as the total power ratio of a band to all bands from delta to gamma (0.5–40 Hz), following the relative power calculation of each band and the ratios of each region. Thus, whether the rsEEG oscillation of relative power (%) relates to the psychometric properties was determined in this study.

### Neuropsychological measures

A questionnaire was administered before EEG recording which comprised four inventories. (1) The DI, which contains 20 items. Scores were weighted from 1 to 4, with a rating of 4 indicating the highest level of depression. Total scores ranged from 20 to 80. (2) The SAI also contained 20 items. Scores were weighted from 1 to 4, where the rating of 4 indicates the highest level of anxiety. Total scores ranged from 20 to 80. (3) The BIS, comprising seven items, probed the degree of anxiety felt when confronted with punishment cues [35]. It was rated on a 4-point Likert scale (range 1–4). A high BIS score was associated with highly negative effects in response to punishments. (4) The IS [38], which contains 16 items. Scores were weighted from 1 to 4, with a rating of 4 indicating the highest level of impulsivity. Total scores ranged from 16 to 64. These inventories were well estimated using the Cronbach’s alpha value and were used for clinical settings to detect and measure the functional manifest.

### Analysis

Descriptive statistical analyses were used for neuropsychological assessments (SAI, DI, BIS and IS) and resting-state band power analysis of delta, theta, alpha, beta and gamma activities (RPV %) at each channel. Consequently, we evaluated the quantitative relationships between psychometric properties and rsEEG rhythms (delta, theta, alpha, beta and gamma powers). Then, the Kolmogorov–Smirnov test (KS test) was applied to determine whether sample data are normally distributed, including frequency bands of the whole brain (%) and the scores of four inventories. Once data revealed a normal distribution, Pearson’s approach was used to evaluate whether correlations existed between the power of the five frequency bands and participants’ neuropsychological scores (SAI, DI, BIS and IS) at the 19 sites accordingly. The significance-level threshold was set at *p* < 0.05. Then, we separately calculated the average correlation coefficients for the 19 sites in each brain wave. Afterwards, average correlation coefficients were used to explore whether any brain wave activity was highly correlated with psychometric properties in perimenopausal women.

This study aimed to understand the association between frequency relative power (delta, theta, alpha, beta and gamma; %) and inventory scores due to previous relevant issues that have not been regarded. First, adequately simple correlations were conducted in the continuous variable using step-by-step reports obtained from five bands and four psychometric inventories at 19 regions. For the purpose of the study, these serial data obtained had no requirement of being spread over multiple comparisons in the elucidated group (or category) difference at this time. However, if inconsistent or particular findings were observed after Pearson’s analysis, we considered additional statistical examinations. Subsequently, a widespread significant relationship was shown in the beta power and impulse strength at most channels, which were inconsistent in others. Therefore, a simple linear regression was conducted to test and illustrate the predictive function of the entire beta oscillation power (%) in four inventories. All statistical analyses were conducted using SPSS version 26.0.

## Results

### Descriptive statistics

Means and standard deviations (mean ± SD) of participants’ scores for the DI AI, BIS and IS were 35.21 ± 5.81, 31.57 ± 8.37, 20.57 ± 3.16 and 34.71 ± 5.03, respectively. The mean and standard error (SE) of the resting band powers (delta, theta, alpha, beta and gamma activity; %) at each channel are shown in Table 1 accordingly. Results showed entire band powers in 19 regions, including five frequencies and four inventory scores, thus revealing normal distribution (*p* > 0.05, KS test).

**Table 1:** The RPV (Relative Power Value, %) at each channel from rsEEG rhythms in perimenopause women

	Delta		Theta		Alpha		Beta		Gamma	
	Mean	SE	Mean	SE	Mean	SE	Mean	SE	Mean	SE
FP1	32.03	3.87	32.03	3.87	23.47	3.91	21.37	1.63	8.54	1.66
FP2	33.84	4.10	33.84	4.10	22.21	3.70	21.28	2.00	8.35	1.58
F7	25.64	2.80	25.64	2.80	24.42	3.71	24.89	1.60	11.42	2.26
F3	18.97	2.75	18.97	2.75	29.46	4.49	26.56	1.78	9.47	1.75
Fz	19.99	2.87	19.99	2.87	31.11	4.62	24.87	1.91	7.06	1.37
F4	21.32	2.78	21.32	2.78	29.20	4.28	26.19	2.01	7.79	1.41
F8	33.63	3.51	33.63	3.51	22.02	3.16	22.57	1.95	8.43	1.45
T3	15.95	2.35	15.95	2.35	22.76	4.25	31.84	2.02	18.20	3.33
C3	14.93	2.16	14.93	2.16	29.83	4.55	30.51	2.57	9.96	2.09
Cz	15.21	2.12	15.21	2.12	32.48	4.96	27.95	2.74	6.90	1.27
C4	16.29	2.13	16.29	2.13	29.94	4.35	30.79	2.76	7.70	1.35
T4	19.74	2.71	19.74	2.71	21.91	3.13	32.50	1.81	13.82	2.69
T5	20.78	3.65	20.78	3.65	24.90	3.03	29.86	2.57	13.60	1.65
P3	18.67	3.89	18.67	3.89	27.74	5.19	31.86	3.39	8.60	1.68
Pz	16.02	2.35	16.02	2.35	29.37	4.74	32.28	3.51	6.87	1.19
P4	15.99	2.46	15.99	2.46	27.56	3.97	34.92	3.13	8.02	1.57
T6	14.01	1.80	14.01	1.80	26.56	3.55	35.50	2.40	12.49	2.89
O1	17.69	3.62	17.69	3.62	20.75	3.46	32.27	2.70	17.50	3.26
O2	14.50	2.15	14.50	2.15	22.40	3.32	37.07	2.84	14.22	3.07

The RPV were analyzed at eye close 3 min resting state for each channel. Five selected frequency bands were Delta (0.5–4 Hz), Theta (4–8 Hz), Alpha (8–13 Hz), Beta (13–30 Hz), and Gamma (31–40 Hz). SE: standard error

### Relationship between brain activity and behaviour

**Fig. 2:**
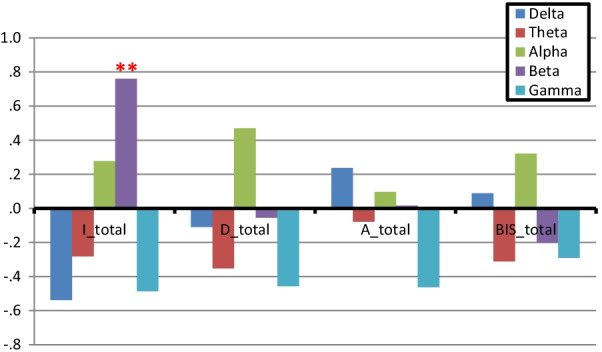
Bands showing the *r* value for the psychometric scores (*r* values were derived from the average of 19 channels). The IS is positively related to the beta power in the cross–correlation analysis. IS, Impulsivity Score; DI, Depression Inventory; SAI, State Anxiety Inventory; BIS, Behavioural inhibition sensitivity scale; **significant at a *p* level of 0.01

Results showed that the resting brain oscillation for delta, theta, alpha, beta and gamma presented different findings using Pearson’s correlation analysis based on the four inventory scores (Table 2). As observed, the IS was positively related to the beta power activity of the symmetrical results for almost all the brain regions (frontal, temporal, central, parietal and occipital regions; *r* = 0.58–0.84, all *p* < 0.05). Contrastingly, DI, AI and BIS scores did not show evident correlations with the delta, theta, alpha, beta and gamma powers (Table 1). Figure 2 shows that the whole-brain resting-beta power may have a specific function in predicting the impulsivity strength in perimenopausal women (*p* < 0.01). We observed that the whole-brain resting activity for delta, theta, alpha, beta and gamma showed no significant correlation with DI, SAI and BIS scores (*p* > 0.05; Fig. 2).

**Table 2:** The Pearson *r* value between the psychological score and band power at each channel

	IS_total					DI_total					SAI_total					BIS_total				
	Delta	Theta	Alpha	Beta	Gamma	Delta	Theta	Alpha	Beta	Gamma	Delta	Theta	Alpha	Beta	Gamma	Delta	Theta	Alpha	Beta	Gamma
FP1	− 0.171	0.044	0.161	0.434	− 0.441	− 0.272	− 0.061	0.444	0.044	− 0.369	0.164	0.276	0.039	− 0.329	− 0.440	− 0.045	0.017	0.215	− 0.073	− 0.345
FP2	− 0.232	0.043	0.138	0.499	− 0.381	− 0.040	− 0.102	0.370	− 0.211	− 0.375	0.404	0.252	− 0.017	− 0.567^*^	− 0.536	0.258	− 0.093	0.069	− 0.352	− 0.281
F7	− 0.294	0.013	0.151	0.662^*^	− 0.347	− 0.389	− 0.065	0.542	0.080	− 0.397	− 0.041	0.153	0.200	0.119	− 0.467	− 0.164	0.156	0.165	− 0.160	− 0.082
F3	− 0.499	− 0.123	0.207	0.740^**^	− 0.345	− 0.253	− 0.333	0.487	0.069	− 0.524	0.170	− 0.031	0.110	0.038	− 0.533	− 0.127	− 0.239	0.276	0.005	− 0.241
Fz	− 0.546	− 0.162	0.292	0.635^*^	− 0.459	− 0.152	− 0.434	0.410	0.054	− 0.466	0.197	− 0.124	0.013	0.094	− 0.385	0.078	− 0.378	0.325	− 0.201	− 0.396
F4	− 0.531	− 0.192	0.234	0.754^**^	− 0.467	− 0.166	− 0.279	0.416	− 0.046	− 0.472	0.274	− 0.025	0.015	− 0.071	− 0.435	0.093	− 0.255	0.259	− 0.218	− 0.308
F8	− 0.418	0.143	0.229	0.600^*^	− 0.434	0.129	− 0.298	0.378	− 0.266	− 0.448	0.408	− 0.077	0.037	− 0.350	− 0.500	0.455	− 0.198	0.085	− 0.509	− 0.383
T3	− 0.127	0.363	0.104	0.414	− 0.452	− 0.193	− 0.083	0.450	− 0.008	− 0.376	0.026	− 0.051	0.170	0.258	− 0.353	− 0.209	0.039	0.008	0.175	0.010
C3	− 0.430	− 0.193	0.080	0.634^*^	− 0.310	− 0.168	− 0.460	0.559^*^	0.032	0.634^*^	0.070	− 0.236	0.249	0.204	− 0.629^*^	0.107	− 0.424	0.238	− 0.030	− 0.197
Cz	− 0.598^*^	− 0.408	0.237	0.603^*^	− 0.388	− 0.111	− 0.562^*^	0.504	− 0.020	− 0.592^*^	0.145	− 0.295	0.125	0.164	− 0.476	0.068	− 0.531	0.442	− 0.102	− 0.540
C4	− 0.683^*^		0.257	0.653^*^	− 0.461	− 0.137	− 0.329	0.462	− 0.104	− 0.524	0.211	− 0.111	0.079	0.050	− 0.498	0.032	− 0.408	0.357	− 0.118	− 0.317
T4	− 0.606^*^		0.244	0.835^**^	− 0.194	− 0.147	− 0.304	0.253	0.178	− 0.073	0.298	− 0.168	− 0.036	0.102	− 0.220	− 0.117	− 0.548	0.014	0.366	0.194
T5	− 0.587^*^		0.552^*^	0.695^**^	− 0.470	− 0.159	− 0.263	0.284	0.126	− 0.147	0.112	− 0.096	− 0.084	0.150	− 0.221	− 0.082	− 0.407	0.496	− 0.106	− 0.300
P3	− 0.358		0.157	0.586^*^	− 0.444	0.018	− 0.570^*^	0.450	− 0.172	− 0.482	0.072	− 0.273	0.172	− 0.003	− 0.402	0.132	− 0.512	0.426	− 0.270	− 0.537
Pz	− 0.617^*^	− 0.612^*^	0.194	0.728^**^	− 0.550	− 0.164	− 0.515	0.523	− 0.107	− 0.479	0.006	− 0.217	0.170	0.012	− 0.318	0.039	− 0.388	0.396	− 0.163	− 0.451
P4	− 0.738^*^	− 0.672^*^	0.339	0.781^**^	− 0.528	− 0.195	− 0.306	0.449	− 0.086	− 0.322	0.028	− 0.092	0.144	− 0.026	− 0.252	− 0.025	− 0.324	0.500	− 0.212	− 0.449
T6	− 0.390	− 0.284	0.435	0.594^*^	− 0.641^*^	0.340	0.031	0.314	− 0.186	− 0.451	0.393	− 0.034	0.046	0.069	− 0.340	0.316	− 0.120	0.454	− 0.359	− 0.397
O1	− 0.365	− 0.433	0.481	0.580^*^	− 0.358	0.225	0.095	0.119	− 0.026	− 0.392	0.358	0.215	− 0.081	0.067	− 0.464	0.209	0.123	0.222	− 0.344	− 0.237
O2	− 0.534	− 0.496	0.403	0.712^**^	− 0.475	0.115	− 0.062	0.318	− 0.045	− 0.343	0.266	− 0.069	0.115	0.072	− 0.338	0.260	0.069	0.349	− 0.343	− 0.270

IS, Impulsivity Score; DI, Depression Inventory; SAI, State Anxiety Inventory; BIS, Behavioural inhibition sensitivity scale

^*^Significant at *p* level < 0.05; **significant at a *p* level of 0.01

After obtaining the entire beta oscillation power, we observed that the Impulse score (IS) was 68% of the total variance in the beta oscillation power (%) using regression analysis (adjusted R^2^ currently = 0.679; unadjusted R^2^ = 0.786). Alternatively, the IS had the maximum variance of all the four scales and it was found to be 68% of the total variance.

## Discussion

Research has not yet clarified the association between spontaneous neural oscillations and neuropsychological evaluations for perimenopausal women. Therefore, this study conducted a quantitative investigation of the associations between rsEEG low-to-high band activities and psychological assessments of perimenopausal women. Results indicated that the rsEEG beta power can be used to specifically predict the degree of impulsivity (as measured by the IS) in perimenopausal women, unlike other psychological characteristics. It has been reported that as mild cognitive decline exists in middle-aged populations, emotion interacts with and influences cognition or behaviour [39]. Furthermore, the beta band expressed a strong relationship with predicted impulses. These findings provide a potential implication for the preclinical or clinical evaluations of perimenopausal women.

Our results indicated a significant positive correlation between the rsEEG beta power and impulsivity (*p* < 0.01; Fig. 2). No such consistent relationship has been observed among the delta, theta, alpha and gamma powers. Previous studies have reported that beta activity was related to symptoms of brain over-arousal, such as anxiety, obsessiveness, sleeping difficulties and hyperactivity in adults [40–43]. Similarly, the IS questionnaire assessed a sense of urgency, premeditation, perseverance and sensation seeking, all of which are factors for impulsive behaviour [44, 45]. Moreover, impulsivity is defined as an individual’s tendency to behave without forethought or regard for the consequences. Studies have revealed that impulsivity is associated with spindling beta activity in clinical conditions, such as attention-deficit/hyperactivity disorder, epilepsy or psychosis and gambling disorders, including during hallucinations [46–48]. Studies providing this evidence have considered employing task-based methods, such as Go/NoGo and stop-signal tasks. However, the brain is constantly active, even at rest without external stimuli [49, 50]. No report has discussed the association between the degree of impulsivity and rsEEG in perimenopausal women. Here, the association between four self-reported psychology inventories across broad frequency bands was evaluated. Subsequently, we highlighted that the intrinsic beta power signature had an important correlation with the degree of impulsivity, making it a potential biomarker for impulsivity in perimenopausal women.

The main finding regarding the relation between spontaneous brain activity and scores of psychological tests was that the default beta power was positively related to IS. This phenomenon was symmetrically exhibited at 16 channels (*r* = 0.58 to 0.84, all *p* < 0.05), with a maximum correlation coefficient (*r* value) at the T4 channel (*r* = 0.84). T4 mainly reveals activity in the middle temporal gyrus [51], connected by regional circuits with the amygdala, hippocampus, superior temporal gyrus, the occipitobasal cortex and orbital gyrus. Additionally, the anatomical and functional organisation of the temporal lobe involves impulse inhibition functions underlying trait impulsivity [52]. Moreover, the temporal lobe communicates with the hippocampus, playing a key role in forming impulsivity. These features are proposed to explain our finding that the maxima correlation coefficient of beta power, with the degree of impulsivity in perimenopausal women, was at the T4 region.

In this study, although other age groups were not included in the comparison of the power of different spectrums, extensive correlation comparisons were made for different psychometric scales. From this preliminary result, it is evident that the indicators of IS represented 68% of the total variance in the entire brain beta oscillation power (%) using regression analysis (adjusted R^2^). Such information has not been reported previously for perimenopausal women, using the non-invasive brain wave device. Impulsiveness is a personality trait whose force can be modulated through hormone fluctuations. These hormonal alterations involve prefrontal-subcortical neurocircuitry modulatory activities that result in an imbalanced contribution of the sub-thalamic nucleus associated with irritability and impulse control disorders [16, 45, 53]. Furthermore, emotions shape behaviour. Inappropriate impulsiveness relates to the anger emotion, which may lead to adverse behaviour such as suicide or violence and interrupt social function. Exploring a reliable indicator for reflecting the intensity of impulses in middle-aged older women is important. Notably, the precise reappraisal of IS or other psychological matters for perimenopausal women also accounts for the advancement in research requirements for clinical or neuroscience researchers. The present study presents a novel academic view on impulsive-EEG biomarkers in the course of menopause transition. For evaluating perimenopause emotional vulnerability, tracking band power modulation through various interventions—such as neurofeedback or meditation for psychological well-being across the lifespan—should be considered in improving women’s health.

We analysed the RPV of frequency bands in each channel using whole-brain averages, not conducting derived analyses of these spectrum bands, such as their spatial coherence or asymmetry. The strength of our approach was that 20–45 epochs were obtained for each band in the statistical analysis, which helped determine the effective size and resulted in a high-reliability estimate. Furthermore, apart from the correlation between the beta power and IS score, the cross-correlational findings of this study identified no neurophysiological biomarker for psychological characteristics in perimenopausal women. These observations further indicate that the quantitative EEG methodology has discriminative power and offers objective and biological insight into cognitive processes, as our study results revealed no significant relationship between delta, theta, alpha, and gamma powers and the SAI, DI, and BIS scores in perimenopausal women. Such a result could encourage clinical care professionals to work on a brain evidence-based manner in the health promotion during the early ageing stages of perimenopausal women.

This study has several limitations. First, we did not address the power spectral density of each band or conduct functional connectivity analysis. Furthermore, we did not apply multiple comparisons due to the fact that the dataset collected was continuous variable, and we had no means of converting these values to categories. Finally, changes in ovarian hormone production affected various neuropsychological health outcomes. Conceivably, however, this study provides more advanced analysis perspectives for future investigations.

## Conclusion

This pilot study shows that the rsEEG beta power specifically predicts impulsivity in perimenopausal women, proposing it as a potential biomarker to assist future research on neurobehavioural estimations in perimenopausal women.

## Data Availability

The datasets used and analyzed during the current study are available from the corresponding author on reasonable request.

## Abbreviations

BIS: Behavioural Inhibition Scale
DI: Depression Inventory
IS: Impulsive Behaviour Scale
RPV: Relative power value
SAI: State Anxiety Inventory
