# Correction to: Association between resting-state EEG oscillation and psychometric properties in perimenopausal women

**DOI:** 10.1186/s12905-022-01820-z

**Published:** 2022-06-16

**Authors:** Ren-Jen Hwang, Hsiu-Chin Hsu, Lee-Fen Ni, Hsin-Ju Chen, Yu-Sheun Lee, Yueh-O. Chuang

**Affiliations:** 1 Department of Nursing, Chang Gung University of Science and Technology, Taoyuan City, 33303 Taiwan, ROC; 2 Department of Nursing, Chang Gung Memorial Hospital, Linkou, Taoyuan City, 33303 Taiwan, ROC; 3 Graduate Institute of Gerontology and Health Care Management, Chang Gung University of Science and Technology, Taoyuan City, 33303 Taiwan, ROC; 4 Department of Internal Medicine, Chang Gung Memorial Hospital, Taoyuan City, 33303 Taiwan, ROC

## Correction to: BMC Women’s Health (2022) 22:149 10.1186/s12905-022-01729-7

Following the publication of the original article [1], the wrong figure appeared as Fig. 1; the Fig. 1 should have appeared as shown below.

**Fig. 1:**
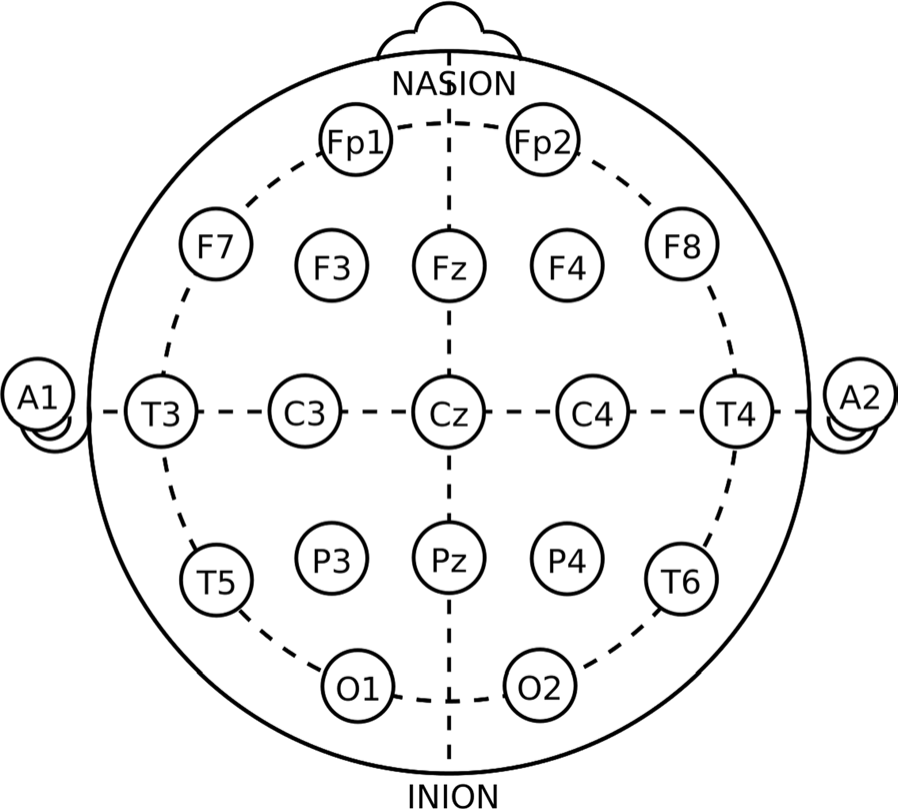
Electrode placement based on the International 10–20 System. The 19 sites: Fp1, Fp2, F3, F4, F7, F8, Fz, C3, C4, Cz, T3, T4, T5, T6, P3, P4, Pz, O1, and O2 within the standard 10–20 system, covering symmetrical brain areas; the anterofrontal, frontal, anterotemporal, temporal, posterior temporal, central, parietal, and occipital regions, are shown

In addition, In Table 2 there are missing numbers in theta column; the Table 2 should have appeared as shown below.

**Table 2:** The Pearson *r*-value between the psychological score and band power at each channel

	IS_total					DI_total					SAI_total					BIS_total				
	Delta	Theta	Alpha	Beta	Gamma	Delta	Theta	Alpha	Beta	Gamma	Delta	Theta	Alpha	Beta	Gamma	Delta	Theta	Alpha	Beta	Gamma
FP1	−0.171	0.044	0.161	0.434	−0.441	−0.272	−0.061	0.444	0.044	−0.369	0.164	0.276	0.039	−0.329	−0.440	−0.045	0.017	0.215	−0.073	−0.345
FP2	−0.232	0.043	0.138	0.499	−0.381	−0.040	−0.102	0.370	−0.211	−0.375	0.404	0.252	−0.017	−0.567*	−0.536	0.258	−0.093	0.069	−0.352	−0.281
F7	−0.294	0.013	0.151	0.662*	−0.347	−0.389	−0.065	0.542	0.080	−0.397	−0.041	0.153	0.200	0.119	−0.467	−0.164	0.156	0.165	−0.160	−0.082
F3	−0.499	−0.123	0.207	0.740**	−0.345	−0.253	−0.333	0.487	0.069	−0.524	0.170	−0.031	0.110	0.038	−0.533	−0.127	−0.239	0.276	0.005	−0.241
Fz	−0.546	−0.162	0.292	0.635*	−0.459	−0.152	−0.434	0.410	0.054	−0.466	0.197	−0.124	0.013	0.094	−0.385	0.078	−0.378	0.325	−0.201	−0.396
F4	−0.531	−0.192	0.234	0.754**	−0.467	−0.166	−0.279	0.416	−0.046	−0.472	0.274	−0.025	0.015	−0.071	−0.435	0.093	−0.255	0.259	−0.218	−0.308
F8	−0.418	0.143	0.229	0.600*	−0.434	0.129	−0.298	0.378	−0.266	−0.448	0.408	−0.077	0.037	−0.350	−0.500	0.455	−0.198	0.085	−0.509	−0.383
T3	−0.127	0.363	0.104	0.414	−0.452	−0.193	−0.083	0.450	−0.008	−0.376	0.026	−0.051	0.170	0.258	−0.353	−0.209	0.039	0.008	0.175	0.010
C3	−0.430	−0.193	0.080	0.634*	−0.310	−0.168	−0.460	0.559*	0.032	−0.634*	0.070	−0.236	0.249	0.204	−0.629*	0.107	−0.424	0.238	−0.030	−0.197
Cz	−0.598*	−0.408	0.237	0.603*	−0.388	−0.111	−0.562*	0.504	−0.020	−0.592*	0.145	−0.295	0.125	0.164	−0.476	0.068	−0.531	0.442	−0.102	−0.540
C4	−0.683*	−0.388	0.257	0.653*	−0.461	−0.137	−0.329	0.462	−0.104	−0.524	0.211	−0.111	0.079	0.050	−0.498	0.032	−0.408	0.357	−0.118	−0.317
T4	−0.606*	−0.051	0.244	0.835**	−0.194	−0.147	−0.304	0.253	0.178	−0.073	0.298	−0.168	−0.036	0.102	−0.220	−0.117	−0.548	0.014	0.366	0.194
T5	−0.587*	−0.406	0.552*	0.695**	−0.470	−0.159	−0.263	0.284	0.126	−0.147	0.112	−0.096	−0.084	0.150	−0.221	−0.082	−0.407	0.496	−0.106	−0.300
P3	−0.358	−0.361	0.157	0.586*	−0.444	0.018	−0.570*	0.450	−0.172	−0.482	0.072	−0.273	0.172	−0.003	−0.402	0.132	−0.512	0.426	−0.270	−0.537
Pz	−0.617*	−0.612*	0.194	0.728**	−0.550	−0.164	−0.515	0.523	−0.107	−0.479	0.006	−0.217	0.170	0.012	−0.318	0.039	−0.388	0.396	−0.163	−0.451
P4	−0.738*	−0.672*	0.339	0.781**	−0.528	−0.195	−0.306	0.449	−0.086	−0.322	0.028	−0.092	0.144	−0.026	−0.252	−0.025	−0.324	0.500	−0.212	−0.449
T6	−0.390	−0.284	0.435	0.594*	−0.641*	0.340	0.031	0.314	−0.186	−0.451	0.393	−0.034	0.046	0.069	−0.340	0.316	−0.120	0.454	−0.359	−0.397
O1	−0.365	−0.433	0.481	0.580*	−0.358	0.225	0.095	0.119	−0.026	−0.392	0.358	0.215	−0.081	0.067	−0.464	0.209	0.123	0.222	−0.344	−0.237
O2	−0.534	−0.496	0.403	0.712**	−0.475	0.115	−0.062	0.318	−0.045	−0.343	0.266	−0.069	0.115	0.072	−0.338	0.260	0.069	0.349	−0.343	−0.270

IS: Impulsivity Score; DI: Depression Inventory; SAI: State Anxiety Inventory; BIS: Behavioural inhibition sensitivity scale

*Significant at *p*-level < 0.05; **significant at a *p*-level of 0.01

The original article has been corrected.
